# Trends in Patient Transfers From Overall and Caseload-Strained US Hospitals During the COVID-19 Pandemic

**DOI:** 10.1001/jamanetworkopen.2023.56174

**Published:** 2024-02-15

**Authors:** Sadia H. Sarzynski, Alex G. Mancera, Christina Yek, Ning An Rosenthal, Alex Kartashov, John L. Hick, Steven H. Mitchell, Maniraj Neupane, Sarah Warner, Junfeng Sun, Cumhur Y. Demirkale, Bruce Swihart, Sameer S. Kadri

**Affiliations:** 1 Critical Care Medicine Department, National Institutes of Health Clinical Center, Bethesda, Maryland; 2 Critical Care Medicine Branch, National Heart Lung & Blood Institute, Bethesda, Maryland; 3 PINC-AI Applied Sciences, Premier, Inc, Charlotte, North Carolina; 4 Hennepin Healthcare, Minneapolis, Minnesota; 5 Department of Emergency Medicine, University of Minnesota Medical School, Minneapolis; 6 Department of Emergency Medicine, University of Washington, Seattle

## Abstract

**Importance:**

Transferring patients to other hospitals because of inpatient saturation or need for higher levels of care was often challenging during the early waves of the COVID-19 pandemic. Understanding how transfer patterns evolved over time and amid hospital overcrowding could inform future care delivery and load balancing efforts.

**Objective:**

To evaluate trends in outgoing transfers at overall and caseload-strained hospitals during the COVID-19 pandemic vs prepandemic times.

**Design, Setting, and Participants:**

This retrospective cohort study used data for adult patients at continuously reporting US hospitals in the PINC-AI Healthcare Database. Data analysis was performed from February to July 2023.

**Exposures:**

Pandemic wave, defined as wave 1 (March 1, 2020, to May 31, 2020), wave 2 (June 1, 2020, to September 30, 2020), wave 3 (October 1, 2020, to June 19, 2021), Delta (June 20, 2021, to December 18, 2021), and Omicron (December 19, 2021, to February 28, 2022).

**Main Outcomes and Measures:**

Weekly trends in cumulative mean daily acute care transfers from all hospitals were assessed by COVID-19 status, hospital urbanicity, and census index (calculated as daily inpatient census divided by nominal bed capacity). At each hospital, the mean difference in transfer counts was calculated using pairwise comparisons of pandemic (vs prepandemic) weeks in the same census index decile and averaged across decile hospitals in each wave. For top decile (ie, high-surge) hospitals, fold changes (and 95% CI) in transfers were adjusted for hospital-level factors and seasonality.

**Results:**

At 681 hospitals (205 rural [30.1%] and 476 urban [69.9%]; 360 [52.9%] small with <200 beds and 321 [47.1%] large with ≥200 beds), the mean (SD) weekly outgoing transfers per hospital remained lower than the prepandemic mean of 12.1 (10.4) transfers per week for most of the pandemic, ranging from 8.5 (8.3) transfers per week during wave 1 to 11.9 (10.7) transfers per week during the Delta wave. Despite more COVID-19 transfers, overall transfers at study hospitals cumulatively decreased during each high national surge period. At 99 high-surge hospitals, compared with a prepandemic baseline, outgoing acute care transfers decreased in wave 1 (fold change −15.0%; 95% CI, −22.3% to −7.0%; *P* < .001), returned to baseline during wave 2 (2.2%; 95% CI, −4.3% to 9.2%; *P* = .52), and displayed a sustained increase in subsequent waves: 19.8% (95% CI, 14.3% to 25.4%; *P* < .001) in wave 3, 19.2% (95% CI, 13.4% to 25.4%; *P* < .001) in the Delta wave, and 15.4% (95% CI, 7.8% to 23.5%; *P* < .001) in the Omicron wave. Observed increases were predominantly limited to small urban hospitals, where transfers peaked (48.0%; 95% CI, 36.3% to 60.8%; *P* < .001) in wave 3, whereas large urban and small rural hospitals displayed little to no increases in transfers from baseline throughout the pandemic.

**Conclusions and Relevance:**

Throughout the COVID-19 pandemic, study hospitals reported paradoxical decreases in overall patient transfers during each high-surge period. Caseload-strained rural (vs urban) hospitals with fewer than 200 beds were unable to proportionally increase transfers. Prevailing vulnerabilities in flexing transfer capabilities for care or capacity reasons warrant urgent attention.

## Key Points

**Question:**

Were hospitals able to increase outgoing patient transfers over time and amid surging caseloads during the COVID-19 pandemic?

**Findings:**

In this cohort study of 681 US hospitals, fewer than usual outgoing transfers to acute care hospitals occurred during periods of high caseloads throughout the COVID-19 pandemic. At top surging hospitals, outgoing transfers increased by 15.4% to 19.8% during the third, Delta, and Omicron waves (vs prepandemic times), but increases were limited to small (<200 beds) urban hospitals.

**Meaning:**

Decreased overall patient movement during the pandemic and the inability to increase transfers from small but overcrowded rural hospitals may indicate patient safety and access to care issues that warrant identifying and overcoming barriers to transfer patients for care or capacity during future surges.

## Teaser

This cohort study evaluates trends in outgoing transfers at overall and caseload-strained hospitals during the COVID-19 pandemic vs prepandemic times.

## Introduction

In the US, approximately 1.5 million patients are transferred between acute care hospitals each year.^1,2^ Patients can be transferred for many reasons, including to receive necessary treatments and services only available at other centers. During the COVID-19 pandemic, many hospitals experienced caseload strain, which may have been detrimental to patients,^3,4,5^ making load balancing (ie, interhospital transfer of patients to less burdened hospitals) an attractive strategy.^6,7,8,9^ In the early phases of the pandemic, interhospital transport appeared to occur relatively safely; one study^10^ found decompensation events during emergency medical services transport for acute lower respiratory tract illness to be infrequent and no greater than a prepandemic baseline. During the early stages of pandemic response, the Joint Pandemic Task Force (including the Office of the Assistant Secretary for Preparedness and Response, now the Administration for Strategic Preparedness and Response) and the Federal Emergency Management Agency expeditiously put out guidance for medical operations coordination cells (MOCCs) to facilitate transfers and load balancing.^11^ Understanding how hospitals changed transfer practices in response to overcrowding and how this changed over the course of the pandemic and varied across small, large, urban, and rural hospitals in the US remains unclear and is critical to inform response efforts during future pandemics.

Evidence on acute hospital transfers during the pandemic has been limited to local or narrative reports, surveys, specific patient cohorts (eg, those with stroke or ST-elevation myocardial infarction), and non-US studies.^6,12,13,14,15,16,17^ Most US states have not reported their experience with transfers to date, and among the few that have, the ability to transfer patients appeared to vary substantially across individual US state–based transfer coordination enters.^17,18^ Furthermore, there were media reports of US hospitals making multiple unsuccessful attempts to move patients to other hospitals.^19,20,21,22,23^ Collectively, these observations raise patient access and equity concerns and questions around the practical feasibility of load balancing as a definitive public health strategy during future pandemics. Using a large database of patient encounters at US hospitals, we studied transfer patterns at overall and caseload strained hospitals during the pandemic drawing comparisons from prepandemic times.

## Methods

### Study Design and Data Source

This retrospective cohort study was performed using PINC-AI (formerly Premier, Inc) Healthcare Database, an all-payer, enhanced administrative data set that contains approximately 25% of all inpatient hospitalizations nationwide. This database has been extensively used in clinical research, and details on the database have been reported previously.^5^ Temporal granularity of these deidentified data is limited to the month level to protect the identities of patients and hospitals. Given the investigators’ need for greater granularity to temporally associate caseload surges and transfers, a unique by-day limited data set with daily data was customized for this study by Premier, Inc (eAppendix in Supplement 1). This allowed for the measurement of daily admissions, hospital census, and outgoing transfers while maintaining information on encounter-level characteristics, such as emergency vs elective admission, emergency department vs inpatient discharge, COVID-19 status, and hospital-level characteristics, such as geographic region, hospital size, urbanicity, and academic status (eTable 1 in Supplement 1). Given the use of only deidentified data, the study was deemed exempt from ethics board review and informed consent (45 CFR 46.102).^24^ This study conforms to the Strengthening the Reporting of Observational Studies in Epidemiology (STROBE) reporting guidelines for cohort studies.^25^

### Study Periods

The prepandemic period was defined as January 1, 2019, to February 29, 2020. Each of the pandemic waves was defined as follows: wave 1, March 1, 2020, to May 31, 2020; wave 2, June 1, 2020, to September 30, 2020; wave 3, October 1, 2020, to June 19, 2021; Delta wave, June 20, 2021, to December 18, 2021; and Omicron wave, December 19, 2021, to February 28, 2022.

### Quantifying Acute Care Transfers

**Figure 1.**
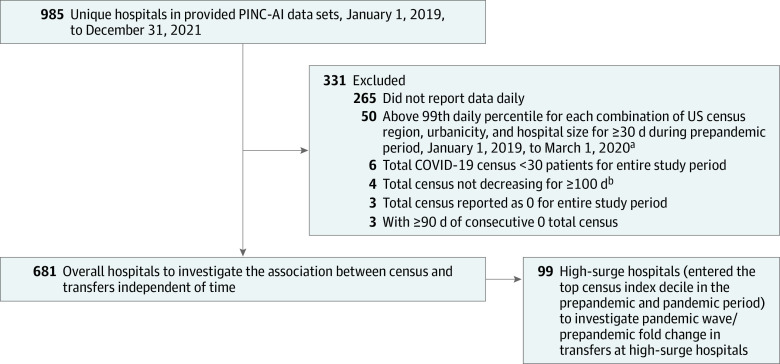
Data Curation Flowchart It was critical to maintain hospitals that reported data daily to reflect accurate trends, which led to the exclusion of 265 hospitals. However, we do not believe that this resulted in a differential bias. ^a^We excluded hospitals with implausible data, which included total census reported as 0 for the entire study period, as well as implausibly high census for prolonged times during the prepandemic period. We also excluded hospitals with very low representation of patients with COVID-19. ^b^Total census steadily increasing from 0 to per-hospital mean is indicative of inconsistent reporting of retroactive data prior to joining PINC-AI Healthcare Database.

Hospitals that continuously reported daily data were included (Figure 1). Outgoing acute care transfers, defined at the hospital of origin as transfers from the emergency department and/or observation or inpatient status to another acute care facility (see eAppendix in Supplement 1), were identified between January 1, 2019, and February 28, 2022. Acuity of the facility receiving transfers from the emergency department, when unspecified, was imputed as acute on the basis of a validation analyses provided in eTable 1 in Supplement 1. This represented 51% of transfers. Weekly outgoing acute hospital transfers were aggregated for each hospital. During the pandemic, transfers of patients with COVID-19 (eAppendix in Supplement 1) were identified according to the presence of an *International Statistical Classification of Diseases and Related Health Problems, Tenth Revision* diagnosis code for COVID-19 (ie, COVID-19 transfers).^26^

### Identifying Surging Hospitals

Hospital daily census index indicated the percentage of licensed beds filled within that hospital on any given day. This was calculated as the daily overall inpatient census (plus new admissions) divided by the prepandemic nominal bed capacity, multiplied by a factor of 100 to obtain a percentage and reported as an mean daily estimate for the week. Nominal bed capacity is reported as ranges to end users of the data set for privacy purposes, and the midpoint of the range was selected for the denominator, as has been done previously with this data source. High-surge hospitals were defined as hospitals that entered the top census index decile (top 10 percentile of census index) at least once in the prepandemic and pandemic period. High-surge weeks were defined as hospital weeks when the hospital was in the top decile of daily census among all hospital weeks in the study.

### Determining Trends in Aggregate Outgoing Transfers

First, weekly trends in all outgoing acute care transfers across all continuously reporting hospitals were analyzed to understand how time and population-level hospital surges may have impacted transfer practices. These data were plotted by COVID-19 status, prepandemic vs pandemic period, and urban vs rural status as well as in relation to the burden of overcrowding represented by the proportion of hospitals that were in the top census index decile in that week (Figure 1).

### Pandemic vs Prepandemic Transfers at Surging Hospitals

Hospital weeks were stratified by prepandemic or pandemic wave–specific periods and by census index categories, respectively.^27^ These categories represented shrinking percentile strata of 0 to 50th, 51st to 75th, 76th to 90th, and 91st to 100th percentiles of the census index. Prepandemic transfer practices for hospitals in the top census index decile were determined and reported by hospital urbanicity and bed capacity.

First, the association between census index and transfer frequency was investigated across all hospitals and across all levels of surge in each period to understand this association independently of time (eTable 2 in Supplement 1). Then, an analogous analysis was performed to understand temporal shifts in outgoing transfer practices specifically at surging hospitals. For this analysis the cohort was limited to high-surge hospitals.^5^ Just as in the first analysis, pairwise transfer comparisons were similarly made against the corresponding hospital’s prepandemic baseline.

### Statistical Analysis

Data analysis was performed from February to July 2023. Linear mixed model regression analysis was conducted between mean weekly transfers and the census index categories to access transfer pattern differences across prepandemic and pandemic waves adjusting for hospital-level characteristics. Mean weekly transfer estimates for each hospital were first logarithm-transformed to meet the normality assumption of the linear models. Although wave-specific estimates were composed of only within-hospital pairwise comparisons in transfers, these were normalized to bed capacity to enable subsequent collation of transfer differences for a given wave across hospitals. Models were adjusted for hospital geographic region, teaching status, bed size, urbanicity, and technological index^5^ to minimize confounding introduced by collation of transfer differences from many hospital types and were also adjusted for calendar month to account for seasonality. Patient level variables were not available to avoid reidentification in highly temporally granular data. The random intercept was also included in the model to account for the associations between observations from the same hospital. Fold change of transfers between prepandemic and each of the pandemic waves and the 95% CIs are calculated under assumption of normal distribution. Hospitals without daily reporting and those with extreme and/or implausible data values reported were excluded. See Figure 1 and the eAppendix in Supplement 1 for details. All statistical tests are 2-sided, with significance set at *P* < .05.

To understand effect modification by hospital size and urbanicity on temporal shifts in transfer practices at surging hospitals, hospitals were first stratified into large urban, large rural, small urban, and small rural hospitals, dichotomized at 200 beds according to bed capacity distributions. Further stratification by technological index was not performed on the basis of observed association with bed capacity. Given that the use of top census index decile to define a high-surge hospital was arbitrary, a sensitivity analysis was performed using hospitals in the top fifth percentile and top fifteenth percentile of the census index to assess for robustness of signal. All analyses were performed using SAS statistical software version 9.4 (SAS Institute).

## Results

**Figure 2.**
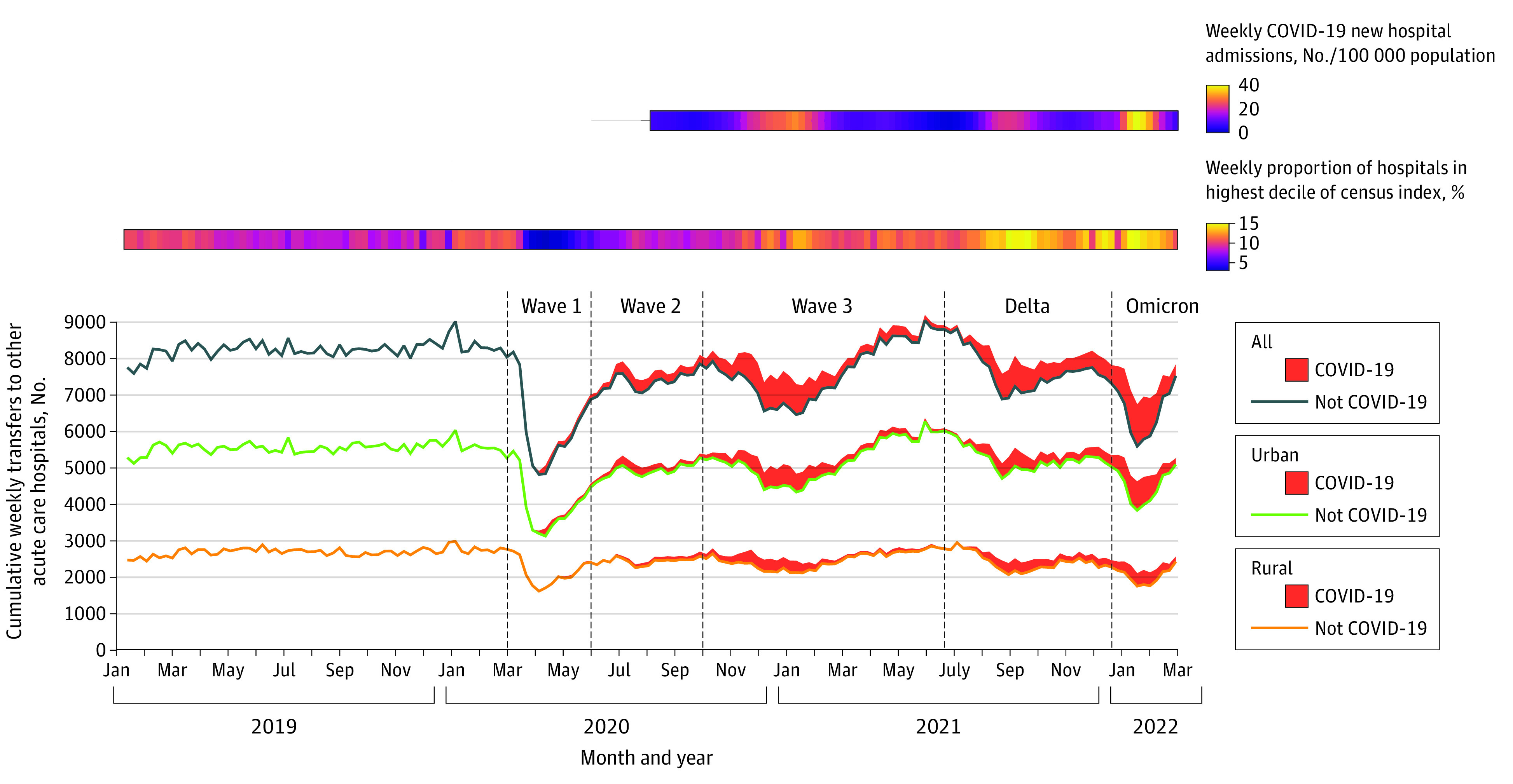
Trends in Cumulative Weekly Transfers to Other Acute Care Hospitals by Urbanicity and COVID-19 Status for 681 US Hospitals Graph shows aggregate weekly outgoing acute-care transfer rates for the overall cohort and broken into urban and rural categories. Transfers of confirmed COVID-19 cases for each group indicated by red shaded area. The first heatmap at the top indicates weekly COVID-19 new hospital admissions per 100 000 population in the US (yellow indicates highest surge).^27^ The second heatmap at the top indicates the weekly proportion of hospitals in the highest decile of the census index (yellow indicates highest surge). These aggregate trends in outgoing transfers were reported for visual interpretation only without statistical tests of significance to avoid overcalling significance given the highly variable aggregate transfers in each week.

Of 985 unique US hospitals in the curated PINC-AI Healthcare Database, 681 hospitals were retained for analysis after exclusions (Figure 1), of which 205 (30.1%) were rural, 476 (69.9%) were urban, 360 (52.9%) were small (<200 beds), and 321 (47.1%) were large (≥200 beds). The weekly trend in aggregate acute care transfers from all 681 hospitals is presented in Figure 2. Overall, 1 305 007 patient transfers occurred to other hospitals during the study period, of which, 426 140 (32.7%) occurred out of rural area hospitals. Compared with the prepandemic period (monthly range, 32 741-37 845 transfers), greater fluctuations were seen in weekly transfers over the course of the pandemic (monthly range, from 22 865 transfers in April 2020 to 38 961 transfers in May 2021). Mean (SD) weekly outgoing transfers per hospital remained lower than the prepandemic mean of 12.1 (10.4) transfers per week for most of the pandemic, ranging from 8.5 (8.3) transfers per week in wave 1 to 11.9 (10.7) transfers per week in the Delta wave.

At 681 study hospitals, even after the initial decrease in transfers in wave 1, subsequent decreases continued to occur during high-surge weeks in each subsequent wave (Figure 2). Conversely, aggregate COVID-19 transfers were consistently higher during high-surge weeks, likely reflecting case mix and indicating that throughout the pandemic fewer than usual patients without COVID-19 were being transferred out during surges. A similar pattern was observed on the aggregate level in both urban and in rural hospitals.

**Figure 3.**
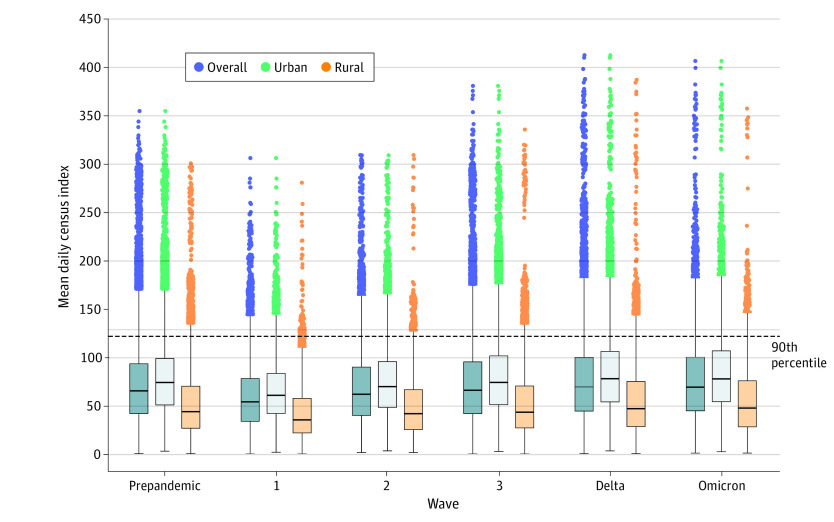
Distribution of Weekly Mean Daily Census Index, During Prepandemic Period and Each Pandemic Wave for 681 US Hospitals Box plots show median (horizontal line), interquartile range (box length), and outliers (dots) of hospital distributions of mean daily census index for weeks in respective periods specified in the figure color coded for hospital type. The mean daily census index above 122 corresponds to the top decile, considered high-surge weeks.

The census index distributions of the 681 overall study hospitals are reported in Figure 3; 148 hospitals (21.7%) entered high-surge weeks during the pandemic, 103 hospitals (15.1%) did so during the prepandemic period, and 99 hospitals (14.5%) did so during both periods. These 99 hospitals represented the population for the primary analysis of transfer trends. There was considerable interhospital and intrahospital variation in high-surge week distributions (eFigures 1 and 2 in Supplement 1), but surge distributions in the study cohort resembled national distributions (Figure 2). Ninety-five of the 99 surging hospitals (96.0%) had 1 or more high-surge weeks during the Delta wave (Table). The peak mean (SD) daily census index for any hospital week over the entire study period was 412.0 (45.2) cases (interpreted as caseload for that week being 412% of the hospital’s bed capacity), which also occurred during the Delta wave (eTable 3 in Supplement 1).

**Table.** Characteristics of 99 US Hospitals in the Highest Decile of the Census Index for at Least 1 Week Prepandemic and at Least 1 Week During the COVID-19 Pandemic

Characteristic	Hospitals, No. (%)					
	Prepandemic^a^	Wave 1^a^	Wave 2^a^	Wave 3^a^	Delta^a^	Omicron^a^
Hospitals in highest decile of census index in each wave^b^	99 (100.00)	83 (83.8)	84 (84.8)	94 (94.9)	95 (96.0)	89 (89.9)
Beds						
<200	59 (59.6)	51 (61.4)	52 (61.9)	56 (59.6)	57 (60.0)	54 (60.7)
≥200	40 (40.4)	32 (38.6)	32 (38.1)	38 (40.4)	38 (40.0)	35 (39.3)
Technological index^c^						
ECMO	39 (39.4)	34 (41.0)	33 (39.3)	38 (40.4)	38 (40.0)	36 (40.4)
Multiple ICUs	8 (8.1)	5 (6.0)	6 (7.1)	7 (7.4)	7 (7.4)	6 (6.7)
Single ICU, CRRT	20 (20.2)	16 (19.3)	18 (21.4)	20 (21.3)	20 (21.1)	19 (21.3)
No ECMO, single ICU, no CRRT	27 (27.3)	24 (28.9)	24 (28.6)	25 (26.6)	27 (28.4)	25 (28.1)
No ECMO, no CRRT, no ICU	5 (5.1)	4 (4.8)	3 (3.6)	4 (4.3)	3 (3.2)	3 (3.4)
Teaching						
No	66 (66.7)	51 (61.4)	59 (70.2)	62 (66.0)	63 (66.3)	60 (67.4)
Yes	33 (33.3)	32 (38.6)	25 (29.8)	32 (34.0)	32 (33.7)	29 (32.6)
Urbanicity						
Rural	20 (20.2)	17 (20.5)	18 (21.4)	18 (19.1)	20 (21.1)	18 (20.2)
Urban	79 (79.8)	66 (79.5)	66 (78.6)	76 (80.9)	75 (78.9)	71 (79.8)
US Census Region						
Midwest	12 (12.1)	11 (13.3)	10 (11.9)	12 (12.8)	12 (12.6)	12 (13.5)
Northeast	17 (17.2)	15 (18.1)	12 (14.3)	17 (18.1)	17 (17.9)	15 (16.9)
South	46 (46.5)	39 (47.0)	41 (48.8)	43 (45.7)	43 (45.3)	41 (46.1)
West	24 (24.2)	18 (21.7)	21 (25.0)	22 (23.4)	23 (24.2)	21 (23.6)
Hospital weeks in highest decile of census index	3888 (100.00)	476 (12.2)	971 (25.0)	2547 (65.5)	1948 (50.1)	782 (20.1)
Beds						
<200	59 (59.6)	51 (61.4)	52 (61.9)	56 (59.6)	57 (60.0)	54 (60.7)
≥200	40 (40.4)	32 (38.6)	32 (38.1)	38 (40.4)	38 (40.0)	35 (39.3)
Technological index^c^						
ECMO	39 (39.4)	34 (41.0)	33 (39.3)	38 (40.4)	38 (40.0)	36 (40.4)
Multiple ICUs	8 (8.1)	5 (6.0)	6 (7.1)	7 (7.4)	7 (7.4)	6 (6.7)
Single ICU, CRRT	20 (20.2)	16 (19.3)	18 (21.4)	20 (21.3)	20 (21.1)	19 (21.3)
No ECMO, single ICU, no CRRT	27 (27.3)	24 (28.9)	24 (28.6)	25 (26.6)	27 (28.4)	25 (28.1)
No ECMO, no CRRT, no ICU	5 (5.1)	4 (4.8)	3 (3.6)	4 (4.3)	3 (3.2)	3 (3.4)
Teaching						
No	66 (66.7)	51 (61.4)	59 (70.2)	62 (66.0)	63 (66.3)	60 (67.4)
Yes	33 (33.3)	32 (38.6)	25 (29.8)	32 (34.0)	32 (33.7)	29 (32.6)
Urbanicity						
Rural	20 (20.2)	17 (20.5)	18 (21.4)	18 (19.1)	20 (21.1)	18 (20.2)
Urban	79 (79.8)	66 (79.5)	66 (78.6)	76 (80.9)	75 (78.9)	71 (79.8)
US Census Region						
Midwest	12 (12.1)	11 (13.3)	10 (11.9)	12 (12.8)	12 (12.6)	12 (13.5)
Northeast	17 (17.2)	15 (18.1)	12 (14.3)	17 (18.1)	17 (17.9)	15 (16.9)
South	46 (46.5)	39 (47.0)	41 (48.8)	43 (45.7)	43 (45.3)	41 (46.1)
West	24 (24.2)	18 (21.7)	21 (25.0)	22 (23.4)	23 (24.2)	21 (23.6)

Abbreviations: CRRT, continuous renal replacement therapy; ECMO, extracorporeal membrane oxygenation; ICU, intensive care unit.

^a^ The prepandemic period is defined as January 1, 2019, to February 29, 2020. Wave 1 is defined as March 1, 2020, to May 31, 2020. Wave 2 is defined as June 1, 2020, to September 30, 2020. Wave 3 is defined as October 1, 2020, to June 19, 2021. The Delta wave is defined as June 20, 2021, to December 18, 2021. The Omicron wave is defined as December 19, 2021, to February 20, 2022.

^b^ Limited to hospitals that were also in the highest decile of census index during prepandemic period.

^c^ The technological index is a multilevel technologic index that stratified hospitals on existing infrastructure for patients with COVID-19.

**Figure 4.**
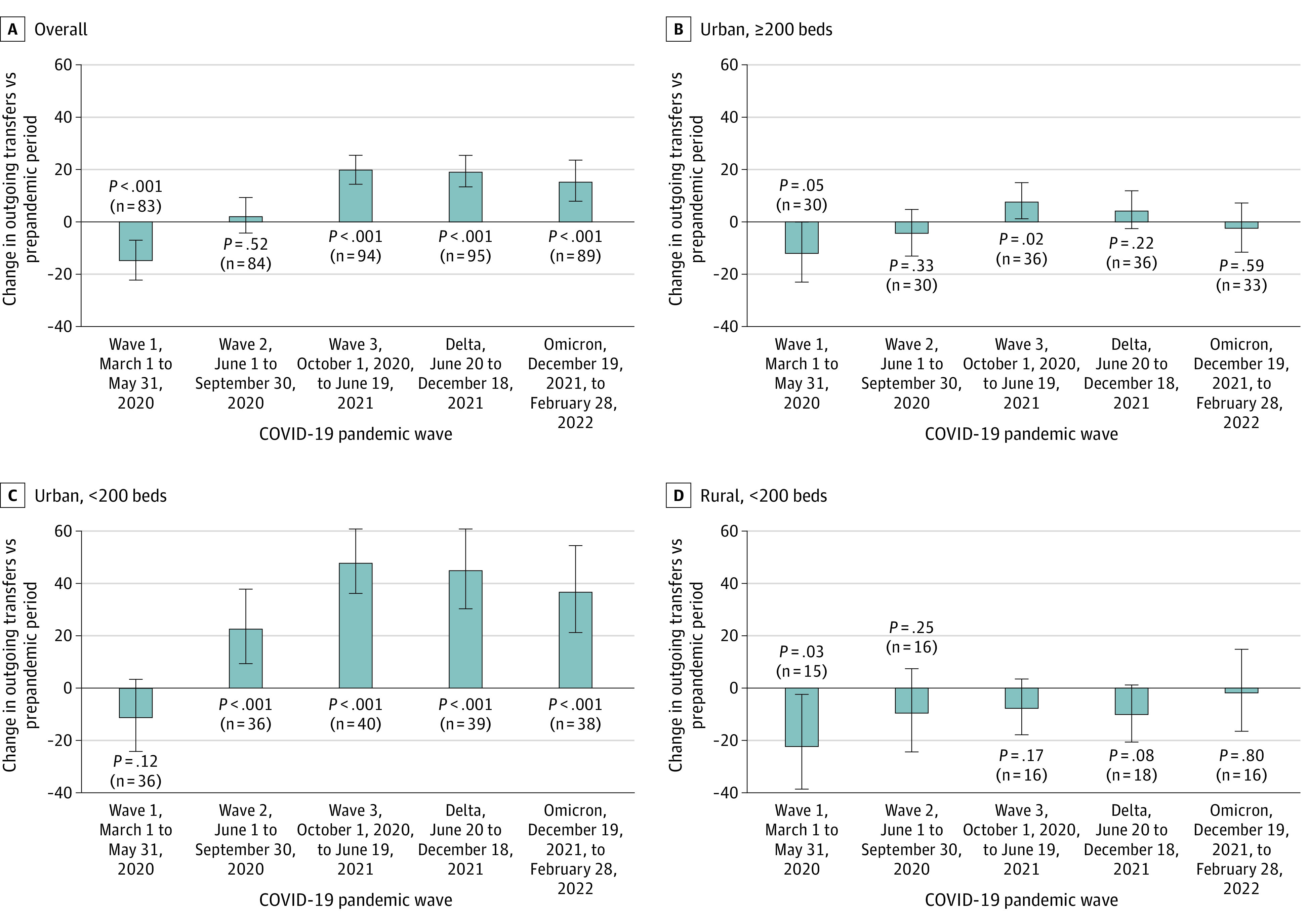
Percentage Change in Outgoing Acute Care Transfers During Each Pandemic Wave Compared With the Prepandemic Baseline for 99 US Surging Hospitals, Showing Overall and Further Stratified by Urbanicity and Hospital Bed Size Fold changes are derived from the linear mixed model, adjusted for seasonality, bed capacity, urbanicity, geographic region, teaching status, and technological index. Increases in transfer rates compared with the prepandemic baseline (fold change) are depicted as above the 0% baseline and decreases below the 0% baseline. Error bars depict the 95% CI. Three large rural hospitals with 200 or more beds were intentionally omitted owing to low count.

The volume of patients appeared to be directly associated with the number of transfers (eTable 2 in Supplement 1). Among the 99 hospitals with prepandemic and pandemic representation of high-surge weeks, transfers decreased, on average, by −15.0% (95% CI ,−22.3% to −7.0%; *P* < .001) during wave 1. Transfers returned to prepandemic baseline during wave 2 (increase of 2.2%; 95% CI, –4.3% to 9.2%; *P* = .52) and then increased beyond prepandemic baseline in wave 3 by 19.8% (95% CI, 14.3% to 25.4%; *P* < .001), which was maintained through the Delta wave at 19.2% (95% CI, 13.4% to 25.4%; *P* < .001) and the Omicron wave at 15.4% (95% CI, 7.8% to 23.5%; *P* < .001) (Figure 4).

Transfer patterns by urbanicity and hospital size were then analyzed for the cohort of 99 surging hospitals. During the prepandemic period, a higher proportion of patients was being transferred at baseline from small (<200 beds) vs large (≥200 beds) hospitals regardless of urbanicity (eFigure 2 in Supplement 1). At 38 large urban hospitals during the pandemic, transfers initially decreased in wave 1 by –12.3% (95% CI, −23.1% to −0.1%; *P* = .048) (Figure 4), with no increases identified compared with the prepandemic period in subsequent pandemic waves. On the other hand, at 41 small urban hospitals, transfers remained higher than prepandemic times during all pandemic waves with a peak increase of 48.0% (95% CI, 36.3% to 60.8%; *P* < .001) observed in wave 3. Only 2 of 99 rural hospitals had more than 200 beds, precluding dedicated analysis of this subgroup. In 18 small rural hospitals, following a decrease in wave 1, no increases in transfers were identified compared with prepandemic times despite high caseloads.

Using alternative cutoffs for defining high-surge weeks, 204 hospitals in the top 15th percentile of census index showed transfer patterns comparable to those seen in the model that used top decile as a cutoff (eFigure 3 in Supplement 1). However, for 91 hospitals within a very high cutoff of the top 5th percentile of census index (census index >95%), fold change distributions of acute outgoing transfers appeared higher than those obtained using a top decile cutoff for the census index.

## Discussion

This cohort study showcases evolutions in patient transfer patterns in US hospitals during the COVID-19 pandemic, providing critical insights with implications on public health policy. It demonstrates the extent to which hospitals transfer capabilities were affected by the stress of overcrowding and how this varied over time, across different hospital types and regions, and in the presence of widespread COVID-19 surges nationally. At 681 continuously reporting US hospitals, we found aggregate transfers to be lower during most of the pandemic compared with prepandemic times, which is the converse of what we would expect during surge conditions. During times of widespread caseload surges, the decrease in non–COVID-19 transfers exceeded the increase in COVID-19 transfers. This finding may represent challenges to transferring patients either for care delivery or load balancing when many hospitals were overloaded at the same time or a reflection of reduced non–COVID-19 admissions. During the first pandemic wave, hospitals transferred fewer than usual patients. This represented a period when surges were localized to some regions, and massive halts in routine and elective care resulted in low census at many US hospitals. Early in the pandemic, news reports emerged showcasing a few hospitals having major surges that were able to eventually transfer large volumes of patients to other hospitals over days.^28,29^ However, on an aggregate basis across hospitals in our study, fewer than usual transfers occurred even during top surging weeks during the first wave. As the pandemic progressed, there was a greater comfort level with managing and transporting patients with COVID-19, and MOCCs emerged in the US for coordinating transports.^17,18^ Hospitals in high-surge weeks in our study displayed a 15.4% to 19.8% increase in transfers during the third, Delta, and Omicron waves, compared with high-occupancy weeks during prepandemic times. However, the transfer patterns during high-surge weeks appeared to be conspicuously different across hospital groups stratified by bed capacity and urbanicity, with growth in transfers being predominantly limited to small urban hospitals.

Although our study is unable to report the explicit indication for transfer or reasons for inability to transfer, our findings weighed against baseline transfer patterns raise questions with important future implications. Larger urban hospitals in our cohort did not appear to increase transfers beyond baseline throughout the pandemic. These hospitals tend to have a wide variety of care settings, subspecialty services, and equipment, and as observed in our study, historically had lesser need to transfer patients elsewhere for higher levels of care. However, in our study, large urban hospitals also did not appear to increase transfers beyond baseline during high-surge weeks. This might indicate low participation or capability in transferring out for capacity generation or load balancing and poses an opportunity for large hospitals to reappraise how they manage high caseloads during future surges given the mortality risk associated with hospital overcrowding.^5^ In our study, small hospitals in urban areas showed up to nearly 50% growth in transfers during high-surge pandemic (vs prepandemic) times. Throughout the pandemic, rural area hospitals during high-surge weeks did not appear to increase transfers beyond a prepandemic baseline. This suggests that for a patient possibly needing transfer for a lifesaving intervention, disproportionately greater barriers to transfer patients may have been encountered in rural vs urban areas. One report from health care leaders of the Arizona, Minnesota, and Washington coordination centers noted that more than 50% of transfer requests during the COVID-19 pandemic originated from rural hospitals.^6^ Others have discussed substantial obstacles encountered when attempting to transfer patients from hospitals located in indigenous rural communities.^15^ One possible explanation is that small urban (vs rural) hospitals are more likely to be affiliated with a health system and its transfer center processes.^29^ Taken together, our findings suggest a sharp contrast between transfer demand and ability for small, rural hospitals during surge-strained conditions, which has implications for potentially increasing preventable mortality for patients and raises concerns about equity and access.

A major barrier to transfer may have been limited capacity, including intensive care unit bed availability and/or staffing constraints, at usual referral and/or tertiary care centers.^30^ The Flex Monitoring Team, a consortium of researchers from 3 US states funded by the Federal Office of Rural Health Policy, performed a survey^31^ of 155 rural critical access hospitals, both system-based and independent, and found that 92% of respondents indicated that the largest barrier for outbound transfers was finding a hospital to accept the transfer or general issues with bed availability; 78% of respondents in the same survey reported their farthest outbound transfers were more than 100 miles. Ground and air transportation availability may have been an additional barrier, as emergency medical services staffing shortages have been described^32,33^; however, a simulation study^34^ also postulated that the number of ambulances may not have been a limiting factor in the US. Interfacility transports are time-consuming processes (particularly when they go beyond usual referral partners), which require coordination and utilization of system resources.^35^ MOCCs successfully emerged at various time points throughout the pandemic but may not have been accessible to many regions where their use may have been critical for transfer coordination.^11,36,37^ Factors besides location, such as MOCC resources, policies, and authority, may have also limited their functionality because some were limited to exclusively transferring patients with COVID-19, some did not have a way to prioritize transfers, and few had the ability to compel acceptance of transfers for emergency conditions.^6^ Another barrier may have been financial and/or regulatory, with reports of several large Southern California hospital systems refusing transfers according to insurance status, a finding that is likely not confined to that area.^21^ Lack of insurance was associated with lower odds of transfer in a study of patients with ST-elevation myocardial infarction.^38^ Although focus is often on transfer from smaller to larger facilities, it is possible that larger facilities, although overwhelmed, may have experienced financial and/or regulatory barriers in transferring or repatriating patients to smaller institutions.^39^ Further investigations are needed to understand what barriers existed and how to overcome these barriers to improve patient outcomes for surge-strained hospitals in the future.^40^

### Limitations

Our study has limitations. The data set does not include data on staffing, thus limiting our ability to gauge effective staffed-bed capacity. Information on indication for transfer or whether a hospital belonged to a health system were not available, which would have further enhanced interpretations. Patient-level variables, such as demographic characteristics and insurance status, were not available to include for case-mix adjustment or further evaluation because data had already been disaggregated to the level of hospital day, and further disaggregation may have risked patient reidentification. Overall emergency department volumes are not accounted for in the analysis, but patients admitted from the emergency department were included in surge calculations from the same day. The acuity level of the receiving center was not known for a portion of the transfers, and although validation analyses substantiated imputing their status to acute, some misclassification may have occurred. The use of top decile of the census index to represent high-surge weeks was arbitrary. Interpretation of the sensitivity analysis using alternative surge cutoffs is limited by the finding that at the individual hospital level, higher census was associated with greater transfers.

## Conclusions

In this study of 681 US hospitals, overall fewer than usual patients were transferred to other acute care centers during periods of pandemic surge. At hospitals during high-surge weeks, transfers decreased in the first wave, but ultimately increased above prepandemic baseline during wave 3 through Omicron wave by 15.4% to 19.8%. However, these increases were largely limited to small urban hospitals. Although load balancing might be an effective strategy to reduce surge-related deaths in theory, the lack of increase in transfers from tertiary centers to other hospitals suggests transferring for capacity was not implemented to any substantial degree at these centers. The inability of small rural hospitals to flex their transfer capabilities during high-surge weeks like small urban hospitals does warrant further investigation to identify reasons for this contrast. Surveys and mixed methods and external validation studies, respectively, might enable further insights and establishment of coordination centers where they are most needed. The quality of and access to public health data and its reporting infrastructure must be improved to enable better real-time action so authorities are not flying blind during the next health crisis. Policies posing barriers to transfer must be identified and addressed urgently to maximally leverage load balancing to mitigate surge strain and save lives, particularly in high-risk regions.

## Notes

Supplement 1. **eTable 1.** Imputation of Emergency Department Discharges to Healthcare Facility of Unspecified Acuity **eTable 2.** Transfer Differences Within Increasing Degrees of Surge for 681 US Hospitals **eTable 3.** Peak Average Daily Census Index per Hospital per Wave **eFigure 1.** Heatmap Showing the Proportional Distribution of High Surge Weeks for Study Hospitals, 99 Hospitals **eFigure 2.** Prepandemic Transfer Patterns During High Surge Weeks for 99 High Surge US Hospitals **eFigure 3.** Fold Change in Outgoing Acute Care Transfers During Each Pandemic Wave Compared to the Prepandemic Period Using Alternative Census Index Cutoffs for Defining a High Surge Week
eReference

Supplement 2.
Data Sharing Statement
