# Supplementary material for: Trends in Patient Transfers From Overall and Caseload-Strained US Hospitals During the COVID-19 Pandemic
Source: JAMA Netw Open. 2024 Feb 15;7(2):e2356174. doi: 10.1001/jamanetworkopen.2023.56174 (PMC10870187; doi:10.1001/jamanetworkopen.2023.56174)
Supplement: Supplement 1. — eTable 1. Imputation of Emergency Department Discharges to Healthcare Facility of Unspecified Acuity eTable 2. Transfer Differences Within Increasing Degrees of Surge for 681 US Hospitals eTable 3. Peak Average Daily Census Index per Hospital per Wave eFigure 1. Heatmap Showing the Proportional Distribution of High Surge Weeks for Study Hospitals, 99 Hospitals eFigure 2. Prepandemic Transfer Patterns During High Surge Weeks for 99 High Surge US Hospitals eFigure 3. Fold Change in Outgoing Acute Care Transfers During Each Pandemic Wave Compared to the Prepandemic Period Using Alternative Census Index Cutoffs for Defining a High Surge Week eReference [file jamanetwopen-e2356174-s001.pdf]

Supplemental Online Content

Sarzynski SH, Mancera A, Yek C, et al. Trends in patient transfers from overall and caseload-strained US hospitals during the COVID-19 pandemic. *JAMA Netw Open*. 2024;7(2):e2356174. doi:10.1001/jamanetworkopen.2023.56174

eTable 1. Imputation of Emergency Department Discharges to Healthcare Facility of Unspecified Acuity

eTable 2. Transfer Differences Within Increasing Degrees of Surge for 681 US Hospitals

eTable 3. Peak Average Daily Census Index per Hospital per Wave

eFigure 1. Heatmap Showing the Proportional Distribution of High Surge Weeks for Study Hospitals, 99 Hospitals

eFigure 2. Prepandemic Transfer Patterns During High Surge Weeks for 99 High Surge US Hospitals

eFigure 3. Fold Change in Outgoing Acute Care Transfers During Each Pandemic Wave Compared to the Prepandemic Period Using Alternative Census Index Cutoffs for Defining a High Surge Week

eReference

This supplemental material has been provided by the authors to give readers additional information about their work.

© 2024 Sarzynski SH et al. *JAMA Network Open.*

eAppendix. Supplemental Methods

Data Curation Steps

Custom data extracts were provided by Premier, Inc to investigators by the data provider granting access to their large file transfer site MoveIT by virtue of a data use agreement [No.75N90020P00477] between the National Institutes of Health and Premier Inc.

The curated datasets, accessed on August 25, 2022, contains daily counts in the categories described below, for patients with specific admit and discharge codes for the period from January 1, 2019, through October 31, 2021. Two datasets were provided, one with overall daily counts of all patients and another for daily counts of patients diagnosed with COVID-19 on arrival. Premier conducted initial data cleaning, removing <0.5% of hospitals with data errors.

The Premier provided variables used in this study are:

- TOTAL_CENSUS, defined as the daily number of inpatients with a billing charge.
- DISCH_ACUTE, defined as the daily number of inpatients discharged to another acute care facility.
- ER_TRANSF_TO_OTHER, defined as the daily number of outpatients with an admit type of “Emergency" or "Urgent" and a discharged to another acute care facility.
- ER_TRANSF_TO_UNCLEAR, defined as the daily number of outpatients with an admit type of “Emergency" or "Urgent" with an unclear acuity of discharge.
- ER_DEATH, defined as the daily number of outpatients with an admit type of “Emergency" or "Urgent" who died or were discharged to hospice.

The Census Index is defined as the daily sum of TOTAL_CENSUS, ER_TRANSF_TO_OTHER, ER_TRANSF_TO_UNCLEAR, and ER_DEATH, which is then normalized to the baseline bed capacity of the hospital, then multiplied by 100 to get more manageable numbers.

Acute outbound transfers are defined as the daily sum of DISCH_ACUTE, ER_TRANSF_TO_OTHER, and ER_TRANSF_TO_UNCLEAR.

Patients with COVID-19 diagnosis were defined using legacy codes for March 2020 and by the presence of ICD-10 Diagnosis Codes U07.1 or J12.82 starting April 1st, 2020.

Intensive care unit (ICU) utilization and type were accessed from the billing file using a previously curated list of billing charges.[1] Based on recommendations from the Centers for Medicare and Medicaid Services, alternative care sites on the hospital campus (e.g., ICU-level care delivered in the hospital parking lot) could be considered a “brick-and-mortar” extension of the hospital. Care delivered at these alternative care sites could be billed for as though it was rendered in the traditional care setting (e.g., the actual ICU). This led to the assumption that our estimate of ICU–level care from billing files potentially captured such care rendered at alternative care sites during the pandemic.

© 2024 Sarzynski SH et al. *JAMA Network Open.*

Mechanical ventilation was defined using the ICD-10 procedure codes 5A1935Z, 5A1945Z, 5A1955Z in conjunction with ICU admission charges on the same day of hospitalization to minimize inclusion of patients chronically ventilated at baseline (under the assumption that mechanical ventilation for acute indications in patients with COVID-19 at alternative care sites are likely to have been captured by the requirement of an ICU charge).

Non-invasive positive pressure ventilation was determine using associated procedure codes and NIPPV use coded for obstructive sleep apnea or obesity hypoventilation syndrome was excluded to mitigate confounding due to chronic use.

The technologic index was created using relevant procedure codes and accommodation charges for extracorporeal membrane oxygenation, and continuous renal replacement therapy and accommodation charges for intensive care unit stays.

Handling of Missing Data

We filtered the Premier datasets, removing 265 (26.9%) hospitals that did report daily, 3(0.3%) hospitals that reported zero census for the entire period, 6 (0.6%) hospitals with very low numbers of COVID-19 patients, 4 (0.4%) hospitals with inconsistent data reporting indicative of joining the Premier network late in the study period, 50 (5.1%) hospitals with extremely high pre-pandemic acute outbound transfer rates, and 3 (0.3%) hospitals with inconsistent data reporting indicative of gaps in data being filled with zeros.

Additional Statistical Methods:

While a more complex random effects structure accounting for temporality could be pursued, we opted to have seasonality in the fixed effects because those effects are of interest given the different waves of pandemic spanning different months of the year. On the other hand, the clustering of encounters in a hospital is viewed more as a statistical nuisance that must be accounted for but not of direct interest and thus a random intercept (exchangeable correlation structure) was considered sufficient.

© 2024 Sarzynski SH et al. *JAMA Network Open.*

eTable 1. Imputation of Emergency Department Discharges to Healthcare Facility of Unspecified Acuity

For a portion of emergency room discharges, the discharge status was “discharge to another healthcare facility” i.e., the acuity level of the receiving facility was unspecified . Here, acuity level was imputed as “acute” based on logic provided below as well as a supportive validation analyses reported below, which was conducted using individual charges for procedures and medications to determine whether acuity distributions resembled emergency department (ED) discharges to acute or subacute facilities. The validation analysis was conducted using in a separate dataset with temporal granularity of encounters up to the month level already accessible to investigators since patient level indicators were unavailable in the customized by-day dataset:

1. Of all 1,115,907 unspecified acuity facility discharges from ED, most [1,008,779 (90.4%)] have a non-facility point of origin (e.g., home, clinic etc.) and few [16,433 (1.5%)] came to the emergency room from facilities specified as “subacute”. It is not plausible that discharges that didn’t originate at subacute facilities went directly to a subacute facility from the emergency room. As such, the planning and time required organize a new placement to a subacute facility for an ED encounter would typically require inpatient admission and case coordination for new placements.
2. As shown in the table below, when patients were discharged from ED to a facility of unspecified acuity, severity indicators of invasive and non-invasive ventilation and vasopressor use during ED stays (without inpatient admission at the same hospital) show this cohort more closely matches ED transfer to “acute” as opposed to ED transfer to “nonacute.” facility

	EMERGENCY DEPARTMENT DISCHARGE TO		
	ACUTE CARE FACILITY	HEATHCARE FACILTY OF UNPSECIFIED ACUITY	SUBACUTE CARE FACILITY
Patients, *n*	123,382	1,115,907	942,566
Vasopressor in ED	2,450 (2.0%)	26,607 (2.4%)	1,157 (0.1%)
NIPPV* in ED	1,274 (1.0%)	13,209 (1.2%)	2,595 (0.3%)
Mechanical ventilation/Intubation in ED	1,169 (0.9%)	17,992 (1.6%)	1,464 (0.2%)

*Non-invasive positive pressure ventilation

© 2024 Sarzynski SH et al. *JAMA Network Open.*

eTable 2: Transfer Differences Within Increasing Degrees of Surge for 681 US Hospitals

The relationship between degree of caseload surge and quantity of transfers was determined within each pandemic wave period. For hospitals that entered a percentile category of the census index for >1 week, the mean of average transfer counts across the surge weeks was used as the hospitals representative estimate of transfers. Distributions of pre-pandemic weekly transfers for top census index decile hospitals were normalized to baseline bed capacity and reported by hospital type to gauge baseline transfer practice patterns. Pairwise within-hospital comparisons of average daily transfers were made to similar averaged estimates from the corresponding census index stratum in the pre-pandemic period.

Time Period	Census Index	Percent change* (CI)	P value
Full Period	0-50 vs 50-75	18.3% (15.1%, 21.5%)	p < 0.0001
	0-50 vs 75-90	31.1% (26.1%, 36.4%)	p < 0.0001
	0-50 vs 90-100	44.9% (37.6%, 52.6%)	p < 0.0001
Pre-pandemic	0-50 vs 50-75	18.4% (14.8%, 22.2%)	p < 0.0001
	0-50 vs 75-90	27.2% (21.8%, 32.8%)	p < 0.0001
	0-50 vs 90-100	31.5% (24.2%, 39.2%)	p < 0.0001
Wave 1	0-50 vs 50-75	24.6% (18.4%, 31.3%)	p < 0.0001
	0-50 vs 75-90	24.0% (15.1%, 33.7%)	p < 0.0001
	0-50 vs 90-100	71.8% (55.6%, 89.7%)	p < 0.0001
Wave 2	0-50 vs 50-75	18.7% (13.2%, 24.5%)	p < 0.0001
	0-50 vs 75-90	35.2% (27.2%, 43.7%)	p < 0.0001
	0-50 vs 90-100	38.5% (28.0%, 49.9%)	p < 0.0001
Wave 3	0-50 vs 50-75	17.2% (13.0%, 21.6%)	p < 0.0001
	0-50 vs 75-90	29.0% (23.1%, 35.3%)	p < 0.0001
	0-50 vs 90-100	48.1% (39.5%, 57.3%)	p < 0.0001
Delta	0-50 vs 50-75	17.3% (12.7%, 22.2%)	p < 0.0001
	0-50 vs 75-90	32.5% (25.8%, 39.5%)	p < 0.0001
	0-50 vs 90-100	40.3% (31.8%, 49.4%)	p < 0.0001
Omicron	0-50 vs 50-75	13.5% (7.4%, 20.0%)	p < 0.0001
	0-50 vs 75-90	39.6% (30.1%, 49.8%)	p < 0.0001
	0-50 vs 90-100	42.5% (31.5%, 54.4%)	p < 0.0001

***All percent changes are in positive direction, thus only increases in transfer rates were noted

Fold changes are derived from the linear mixed model—adjusted for seasonality, bed capacity, urbanicity, geographic region, teaching status, and technological index.

© 2024 Sarzynski SH et al. *JAMA Network Open.*

eTable 3: Peak Average Daily Census Index per Hospital per Wave

Wave	Full Cohort (681 hospitals)*	Hospitals excluded from Model Cohort (582 hospitals)
Pre-Pandemic	354.3	131.9
Wave 1	305.7	144.5
Wave 2	308.9	145.3
Wave 3	380.3	169
Delta	412	190.3
Omicron	406	176.3

The maximum per-hospital weekly average Census Index per wave using the overall cohort (681 hospitals) and hospitals excluded from the model cohort (582 hospitals).
Peak average daily census index per hospital per wave are the same for 99 high surge hospitals.

© 2024 Sarzynski SH et al. *JAMA Network Open.*

eFigure 1: Heatmap Showing the Proportional Distribution of High Surge Weeks for Study Hospitals, 99 Hospitals

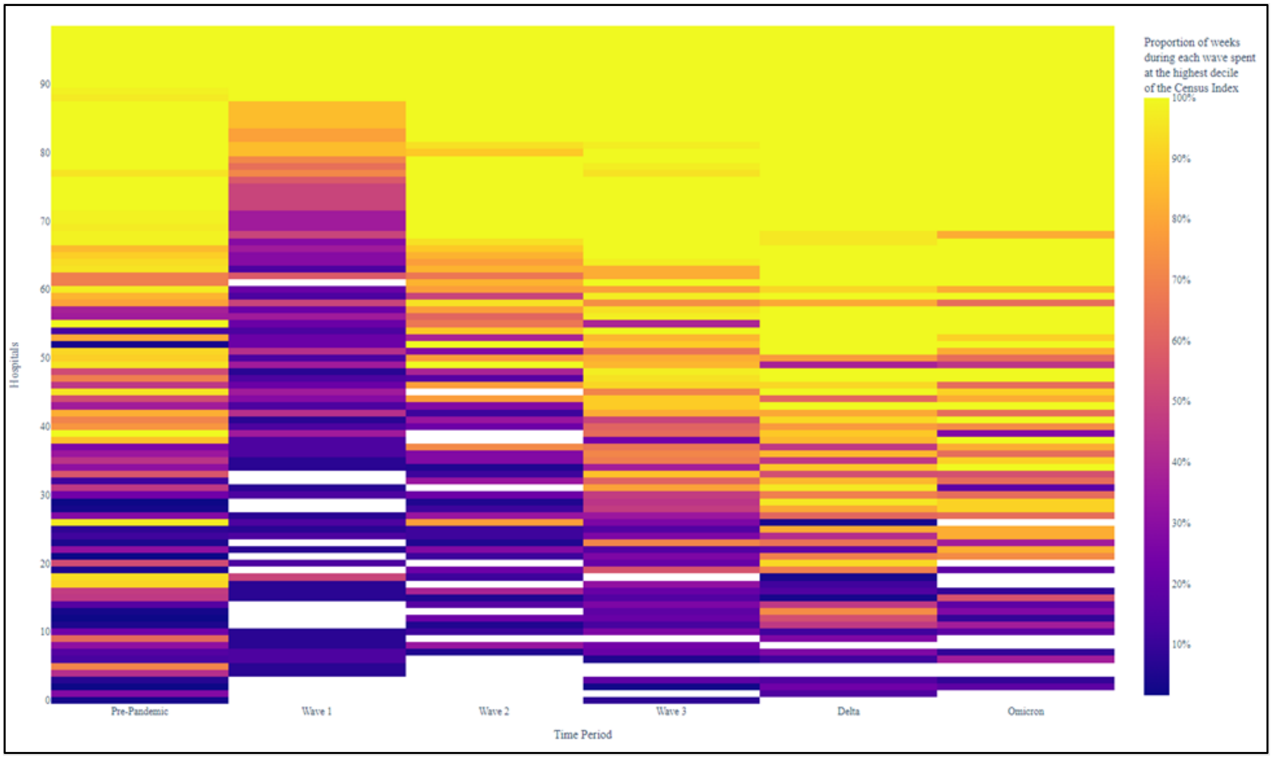

Each row in the heatmap represents a hospital that entered the top Census Index decile at least once during the pre-pandemic and pandemic periods respectively. White cells for any given wave indicate the hospital never entered the top Census Index decile during that wave.

© 2024 Sarzynski SH et al. *JAMA Network Open.*

eFigure 2: Prepandemic Transfer Patterns During High Surge Weeks for 99 High Surge US Hospitals

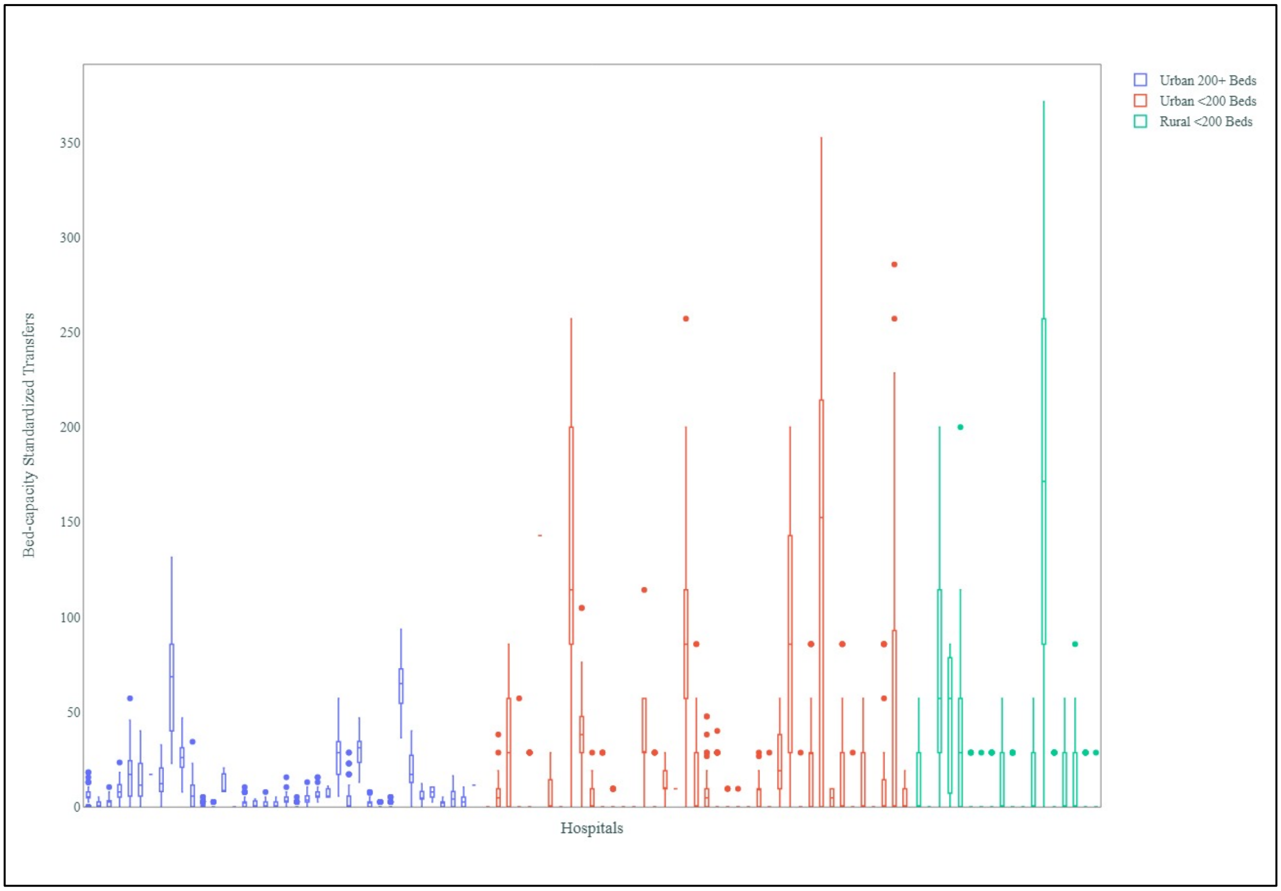

The pre-pandemic (or "baseline") transfer patterns during high surge weeks for the 99 high surge U.S. hospitals are depicted by urbanicity and bed-size. At the pre-pandemic baseline, large, urban hospitals proportionally transferred fewer patients when compared with small urban and small rural hospitals. At baseline, small rural hospitals transfer patients out comparably to small urban hospitals.

Note: weekly transfers are bed-capacity standardized by dividing the estimates by baseline hospital bed capacity to enable comparisons in transfer tendency across hospitals and multiplied by 100 for ease of reporting. The median of standardized weekly transfers (horizontal bar within IQR), and corresponding interquartile range (thick vertical bars), and upper and lower fences (thin vertical bars) defined as the IQR +/- 1.5 times their respective Q3 or Q1, respectively are presented in the figure for hospitals from pre-pandemic weeks spent in high surge weeks (i.e., in the top census index decile) and color coded by hospital type. Data are not shown for the two large (≥200-bed) rural hospitals.

© 2024 Sarzynski SH et al. *JAMA Network Open.*

eFigure 3: Fold Change in Outgoing Acute Care Transfers During Each Pandemic Wave Compared to the Prepandemic Period Using Alternative Census Index Cutoffs for Defining a High Surge Week

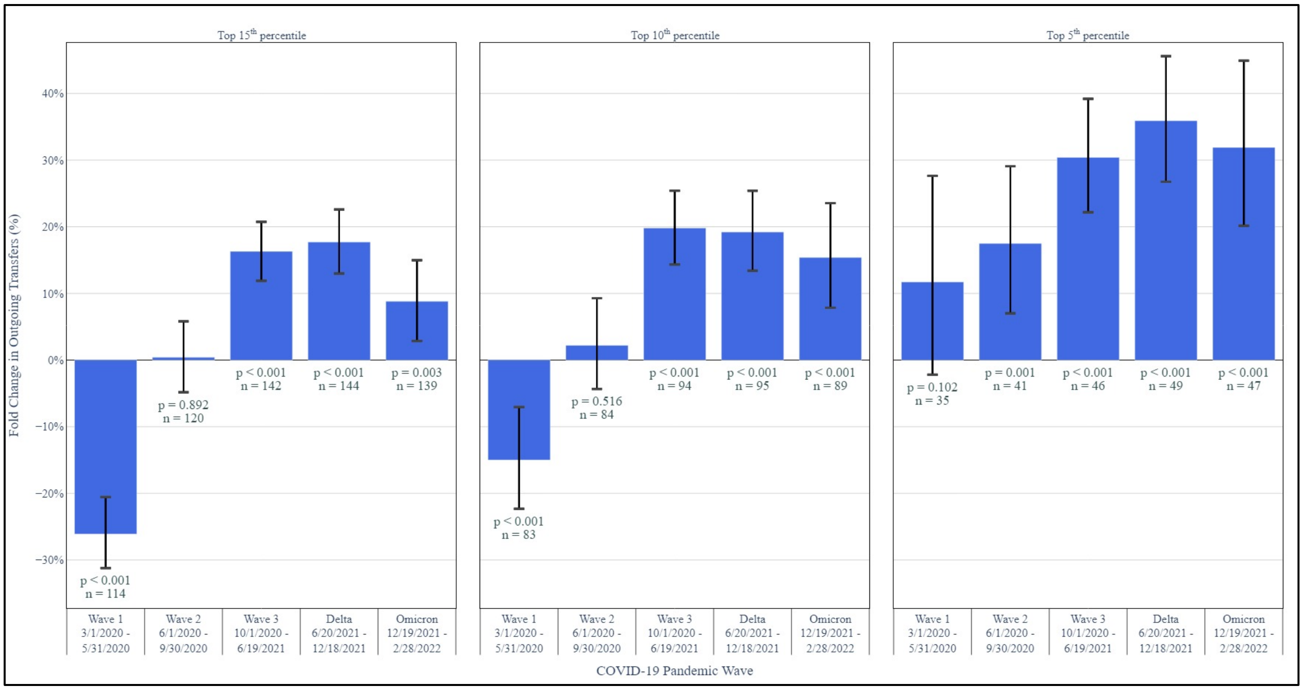

Fold changes are derived from the linear mixed model—adjusted for seasonality, bed capacity, urbanicity, geographic region, teaching status, and technological index.

Increases in transfer rates relative to pre-pandemic baseline are depicted as above the 0% percent baseline and decreases below the 0% baseline. Whiskers depict the 95% confidence interval.

*Pre-pandemic period = 1/1/2019 - 2/29/2020

eReference

1. Kadri SS, Sun J, Lawandi A, et al. Association Between Caseload Surge and COVID-19 Survival in 558 U.S. Hospitals, March to August 2020. *Ann Intern Med*. Sep 2021;174(9):1240-1251. doi:10.7326/M21-1213

© 2024 Sarzynski SH et al. *JAMA Network Open.*
