# Supplementary material for: Trends in Patient Transfers From Overall and Caseload-Strained US Hospitals During the COVID-19 Pandemic
Source: JAMA Netw Open. 2024 Feb 15;7(2):e2356174. doi: 10.1001/jamanetworkopen.2023.56174 (PMC10870187; doi:10.1001/jamanetworkopen.2023.56174)
Supplement: Supplement 2. — Data Sharing Statement [file jamanetwopen-e2356174-s002.pdf]

Data Sharing Statement

Sarzynski. Trends in Patient Transfers From Overall and Caseload-Strained US Hospitals During the COVID-19 Pandemic. *JAMA Netw Open*. Published February 15, 2024. doi:10.1001/jamanetworkopen.2023.56174

Data
Data available: No
